# On-chip Fourier-transform spectrometer based on spatial heterodyning tuned by thermo-optic effect

**DOI:** 10.1038/s41598-019-50947-x

**Published:** 2019-10-10

**Authors:** Miguel Montesinos-Ballester, Qiankun Liu, Vladyslav Vakarin, Joan Manel Ramirez, Carlos Alonso-Ramos, Xavier Le Roux, Jacopo Frigerio, Andrea Ballabio, Enrico Talamas, Laurent Vivien, Giovanni Isella, Delphine Marris-Morini

**Affiliations:** 1 Centre for Nanoscience and Nanotechnology (C2N), CNRS - Université Paris-Sud - Université Paris-Saclay, 91120 Palaiseau, France; 2 L-NESS, Dipartimento di Fisica, Politecnico di Milano, Polo di Como, Via Anzani 42, 22100 Como, Italy; 3 Present Address: Nexdot, 102 Avenue Gaston Roussel, 93230 Romainville, France; 4 Present Address: III-V Lab, 91120 Palaiseau, France

**Keywords:** Mid-infrared photonics, Integrated optics, Infrared spectroscopy

## Abstract

Miniaturized optical spectrometers providing broadband operation and fine resolution have an immense potential for applications in remote sensing, non-invasive medical diagnostics and astronomy. Indeed, optical spectrometers working in the mid-infrared spectral range have garnered a great interest for their singular capability to monitor the main absorption fingerprints of a wide range of chemical and biological substances. Fourier-transform spectrometers (FTS) are a particularly interesting solution for the on-chip integration due to their superior robustness against fabrication imperfections. However, the performance of current on-chip FTS implementations is limited by tradeoffs in bandwidth and resolution. Here, we propose a new FTS approach that gathers the advantages of spatial heterodyning and optical path tuning by thermo-optic effect to overcome this tradeoff. The high resolution is provided by spatial multiplexing among different interferometers with increasing imbalance length, while the broadband operation is enabled by fine tuning of the optical path delay in each interferometer harnessing the thermo-optic effect. Capitalizing on this concept, we experimentally demonstrate a mid-infrared SiGe FTS, with a resolution better than 15 cm^−1^ and a bandwidth of 603 cm^−1^ near 7.7 *μ*m wavelength with a 10 MZI array. This is a resolution comparable to state-of-the-art on-chip mid-infrared spectrometers with a 4-fold bandwidth increase with a footprint divided by a factor two.

## Introduction

Photonics integration in the mid-Infrared (mid-IR) spectral range, and more specifically in the fingerprint region between 5 and 20 *μ*m wavelength has garnered a great interest due to its immense potential for applications in spectroscopy and sensing. The unique vibrational and rotational resonances of molecules at these wavelengths can be used to univocally discern and quantify the molecular composition of a broad variety of gases, liquids or solids, with application in environmental monitoring^1^, astronomy^2^, hazard detection^3^, industrial process control^4^ and non-invasive medical diagnostics^5,6^. While commercial mid-IR spectrometers currently available are often based on an assembly of discrete elements^7^, on-chip photonic integration offer key advantages to develop fast, low-cost, low-power consumption, high-reliability and high performances systems.

A myriad of integrated spectroscopic systems has been demonstrated, based on dispersive devices such as array waveguide gratings (AWG)^8,9^, echelle grating^10,11^ or Fourier transform spectroscopy^12–22^. On-chip Fourier transform spectrometers (FTS) implement simple yet effective calibration algorithms that compensate phase and amplitude impairments, thereby providing a superior robustness against fabrication imperfections, compared to dispersive counterparts. Current on-chip FTS rely on one of the three following operation schemes: i) stationary wave interferometry (SWI), ii) static spatial heterodyne spectroscopy (SHS), and iii) scanning interferometry. In SWI an stationary wave pattern is generated by the interference of two counter- or co-propagating waves^13,17^. This interference pattern is diffracted off-chip and monitored by an array of photodetectors. The resolution of this approach is seriously limited by practical constraints in the photodetector array, e.g. minimum pitch size. In static SHS, an array of interferometers with linearly increasing optical path delays is implemented to yield a spatial interferogram that allows on-chip spectral detection without any moving parts^12,14–16,21^. SHS-based devices can easily implement remarkably large optical path delays which resulted in unprecedented resolutions^14^. In addition, multi-aperture SHS exhibit an increased optical throughput (Jacquinot advantage), which is key for some spectroscopy applications^12^. However, their bandwidth is limited by the minimum optical path difference among consecutive interferometers. Achieving a fine resolution (large optical delay) and broadband operation (small optical delay step) requires an unpractical large number of interferometers. Integrated scanning-interferometry-based spectrometers implement the same approach as bulk optics Michelson interferometers, i.e. the scanning of the optical path delay within the interferometer. In the integrated approach, the scanning of the path difference is achieved by an external tuning which changes either the length or the optical properties of the optical path. A high-resolution spectrometer based on scanning interferometry has recently been demonstrated based on a discrete set of optical path differences dynamically selected by optical switches^20^. Nevertheless, the bandwidth of this device is still limited by the minimum implementable optical path difference. The thermo-optic effect has also been used to scan the optical path delay of single interferometers^18,19,22^. However, the resolution of these devices is limited by the maximum achievable optical delay. Finally, theoretical studies predicted that the bandwidth or resolution of the spectrometer could be improved by an array of thermally-tuned filters^23^.

**Figure 1:**
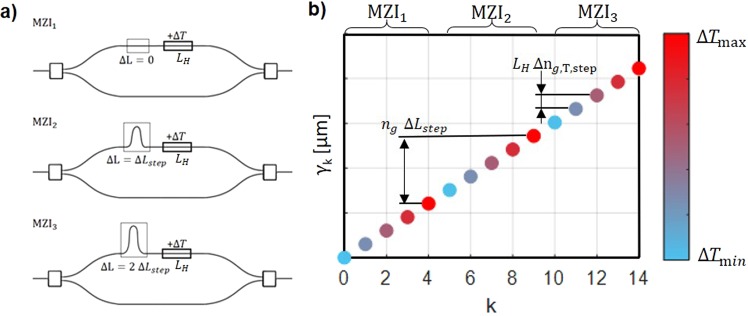
(**a**) Generic schematic of the proposed Fourier transform spectrometer based on spatial heterodyning tuned by thermo-optic effect. (**b**) Schematic representation of the uniform sampling of the optical path delay *γ*, based on thermally-tuned Mach Zehnder array, showing the first 3 MZI with 5 different applied temperatures.

Here, we propose and experimentally demonstrate an alternative approach that gathers in a simple but effective way the advantages of spatial heterodyning and optical path tuning by thermo-optic effect to overcome the bandwidth-resolution tradeoff in conventional counterparts. The proposed approach, depicted in Fig. 1(a), implements an array of Mach-Zehnder interferometers (MZI) with a linearly increasing optical path length difference (Δ*L*) and a common thermal tuned length *L*_*H*_ in each one. The optical delay in in each MZI is changed by applying a thermal shift with a waveguide heater. The output interferogram is formed by recording the output of each interferometer for different temperatures. The resolution of the system is mainly governed by the maximum optical path length difference (Δ*L*_*max*_), while the bandwidth is determined by the minimum thermally-induced optical path delay (${L}_{H}\,\Delta {n}_{g}(T)$). This way, fine resolution and broad operation can be achieved without the need for a large number of interferometers, overcoming the major limitation of conventional SHS-based devices. Based on this new approach, we have implemented a mid-IR spectrometer with Ge-rich SiGe waveguides, experimentally showing a bandwidth of 603 cm^−1^ with a resolution below 15 cm^−1^. State-of-the-art on-chip FTS resolution in the mid-infrared is thus obtained with a 4-fold bandwidth increase.

## Results

### Working principle of FTS based on thermally-tuned spatial heterodyning

The theory and operational principle of integrated spatial heterodyne Fourier transform spectrometers (SHFTS) have been reported previously by Florjanczyk *et al*.^12^. In a typical configuration, a SHFTS is composed of a MZI array with path difference changing by a constant increment across the device.

In this work we propose to generate path delays through a combination of MZI arm length differences together with thermal tuning of the optical path in each MZI. For this purpose, the mathematical model of the MZI array is generalized to the thermally-tuned MZI array. As it is later detailed in Methods section, the resolution *δσ* and bandwidth, also referred as free spectral range (FSR_*σ*_), are given by:

1 $$\delta \sigma =\frac{1}{{\gamma }_{max}}=\frac{1}{\Delta {L}_{max}\,{n}_{g}+{L}_{H}\,\Delta {n}_{g,T,max}},$$

2 $$FS{R}_{\sigma }=\frac{1}{2\,{\gamma }_{step}}=\frac{1}{\Delta {L}_{max}\,{n}_{g}+{L}_{H}\,\Delta {n}_{g,T,max}}\frac{{N}_{T}\,{N}_{L}}{2},$$

where *γ*_*max*_ is the maximal optical path delay difference between MZI arms, *γ*_*step*_ is the sampling interval of the optical path delay difference, *n*_*g*_ is the group index of the guided optical mode, $\Delta {n}_{g,T,max}$ is the highest group index change due to thermal tuning, Δ*L*_*max*_ is the largest arm length unbalance in the MZI array, *N*_*T*_ is the number of different temperatures applied and *N*_*L*_ is the number of MZIs in the array. The largest optical path delay difference *γ*_*max*_ is achieved when the highest temperature change is applied through the heating length *L*_*H*_ and thus producing the largest group index change $\Delta {n}_{g,T,max}$ in the most unbalance MZI of the array, i.e. when the length difference between both arms is Δ*L*_*max*_. According to Nyquist-Shannon theorem, the FSR is inversely proportional to two times the sampling interval *γ*_*step*_ (Eq. (2)). It means that the smaller is the sampling interval, the larger is the bandwidth of the SHFTS. Generally speaking, the thermally-induced phase shift is much lower that the maximal phase shift due to MZI path difference, thus $\Delta {L}_{max}\,{n}_{g}\gg {L}_{H}\,\Delta {n}_{g,T,max}$. Therefore, the resolution of the thermally-tuned FTS is very close to the one of classical FTS, and mainly depends on the path difference of the most unbalanced MZI in the MZI array. On the other hand, the FSR can be largely improved in thermally-tuned FTS. Indeed, the main advantage of introducing thermal tunability in the MZI array is to break the fundamental tradeoff between FSR, resolution and number of MZI, by improving the spectrometer FSR keeping reasonable number of MZI in the array. The fundamental relation FSR = *δσN*_*L*_/2 in classical FTS is thus replaced by FSR = *δσN*_*T*_*N*_*L*_/2 in thermally-tuned FTS, where the number of MZI (*N*_*L*_) is now multiplied by the number of different temperature *N*_*T*_ used in the measurement, considering *γ* points equally spaced.

In order to evaluate the implementation of thermally-tuned FTS, first we consider a uniform sampling of the optical path delay as illustrated in Fig. 1(b). Since much larger path delays are obtained through MZI arm difference length compared to thermal tuning, thermal tuning will be used to sample the spectral region between the transmission peaks of consecutive MZI with different arm lengths. In the following, Δ*L*_*step*_ is the incremental change of path difference across the MZI array and $\Delta {n}_{g,T,step}$ is the incremental change of the group index, related to the incremental change of temperature Δ*T*_*step*_ induced by the heater, following the relation $\Delta {n}_{g,T,step}=\frac{\partial {n}_{g}}{\partial T}\Delta {T}_{step}$. Since for the mid-infrared wavelength range the thermo-optic coefficient can be considered constant with the wavelength^24^, this incremental change of the group index can be also considered constant, too. If we also consider that the first MZI is balanced and, for simplicity, that the temperature varies only for positive values from $\Delta T=0$ to $({N}_{T}-1)\,\Delta {T}_{step}$ for all MZI, *γ* is thus taking ${N}_{T}\times {N}_{L}$ different equally spaced values from ${\gamma }_{min}=0\,{\rm{m}}$ to ${\gamma }_{max}=({N}_{L}-\mathrm{1)}\,{n}_{g}\,\Delta {L}_{step}+({N}_{T}-\mathrm{1)}{L}_{H}\,\Delta {n}_{g,T,step}$.

Considering a uniform sampling of the optical path delay, we can finally deduce the relation between the $\Delta {n}_{g,T,step}$, Δ*L*_*step*_ and *N*_*T*_ as expressed in Eq. (3), where the next temperature increment would correspond with the next MZI with no thermal tuning applied.

3 $${N}_{T}\,{L}_{H}\,\Delta {n}_{g,T,step}={n}_{g}\,\Delta {L}_{step}$$

Δ*T*_*step*_ and the maximal temperature variation Δ*T*_*max*_ are then also given by Eqs (4) and (5).

4 $$\Delta {T}_{step}=\frac{1}{\frac{\partial {n}_{g}}{\partial T}}\frac{{n}_{g}\,\Delta {L}_{step}}{{N}_{T}{L}_{H}},$$

5 $$\Delta {T}_{max}=({N}_{T}-\mathrm{1)}\Delta {T}_{step}=({N}_{T}-\mathrm{1)}\frac{1}{\partial {n}_{g}/\partial T}\frac{{n}_{g}\,\Delta {L}_{step}}{{N}_{T}\,{L}_{H}},$$

Furthermore, it can be noted that as it is the case for classical FTS, thermally-tuned FTS can suffer of deviations from the ideal design due to phase errors, but these errors can be eliminated by a proper calibration procedure^14^. Instead of using a cosine transform, a spectral retrieval method is used based on a set of linear equations and on the measurement of the transfer matrix of the spectrometer. To this end, the transfer matrix T is first built by scanning the input wavelength within the spectral range of the spectrometer while recording the output of each MZI. The output of all MZI is also recorded for each temperature. With all these measurements a ${N}_{T}{N}_{L}\times M$ matrix is obtained, where M is the number of different measured wavelengths. When an unknown signal is set as the input spectrum, the measured interferogram is given by $I({\gamma }_{k})=S\times T$, where S is the input spectrum. Therefore, it is possible to retrieve any unknown input spectrum S by multiplying the measured interferogram by *T*^+^, the pseudo-inverse of T^16^. Moreover, as it is later demonstrated in this work, with this method it is even possible to retrieve a non-uniform sampled spectrum. It must be also pointed out that other procedures such as Elastic-D1 method^20^ or *l*1-norm minimization^25^ can be applied to enhance the performance of the retrieval process.

### Mid-IR SiGe FTS based on thermally-tuned spatial heterodyning

Up to now, most of the on-chip FTS demonstrations have used the silicon-on-insulator (SOI) platform, which provides a mature technology compatible with near-infrared and short-wave-infrared (SWIR) wavelength range. Thus, state-of-the-art integrated SHFTS have shown operation mainly at 1.55 *μ*m^14,18,20^ and in the SWIR spectral region, below 4 *μ*m wavelength. However, the development of photonic platforms dedicated to longer MIR wavelengths has recently witnessed a burst of research activity^26–37^. Germanium-rich silicon-germanium (Ge-rich SiGe) has emerged as a promising integrated platform exhibiting a wide transparency range^38^. A linearly graded SiGe layer allows a smooth transition between pure silicon and Ge-rich material that minimizes the threading dislocation density due to lattice mismatch, while confining the optical mode in the upper part of the waveguide by refractive index gradient. The recent experimental demonstration of a MIR SHFTS^21^ provides a promising departing point to develop new deep MIR spectroscopic systems. In that previous work an experimental resolution better than 15 cm^−1^ was obtained with a FSR of 132 cm^−1^. Interestingly, the operating frequency range was much larger as it reached 800 cm^−1^. All these compelling features make the Ge-rich SiGe an excellent platform for developing a broadband thermally-tuned mid-IR FTS. As a main objective we target to increase the device FSR, without degrading the resolution nor increasing the device area.

To demonstrate the advantage and viability of the thermally-tuned FTS, we consider first a device similar to the one reported by Q. Liu *et al*.^21^, where broadband operation was demonstrated (800 cm^−1^), but the device was limited by its FSR. We will numerically evaluate the advantage of thermally-tuning this device and demonstrate its performance enhancement. Therefore, an array of 10 MZI is considered (9 unbalanced and 1 balanced MZI), with Δ*L*_*step*_ of 18.8 *μ*m, which means half of the devices reported by Q. Liu *et al*.^21^, but with a 3 mm-long heater located in the shortest arm of each MZI. In terms of thermo optic coefficient, as the optical mode is confined in Ge-rich SiGe materials, the thermo-optic coefficient of Ge is used, and it is considered constant within the operational wavelength range with a value of $4.1\times {10}^{-4}\,{K}^{-1}$. Indeed, the variation is estimated to be lower than $0.1\times {10}^{-4}\,{K}^{-1}$ in the wavelength rang of interest, i.e. from 5.5 to 8.5 *μ*m^24^.

**Figure 2:**
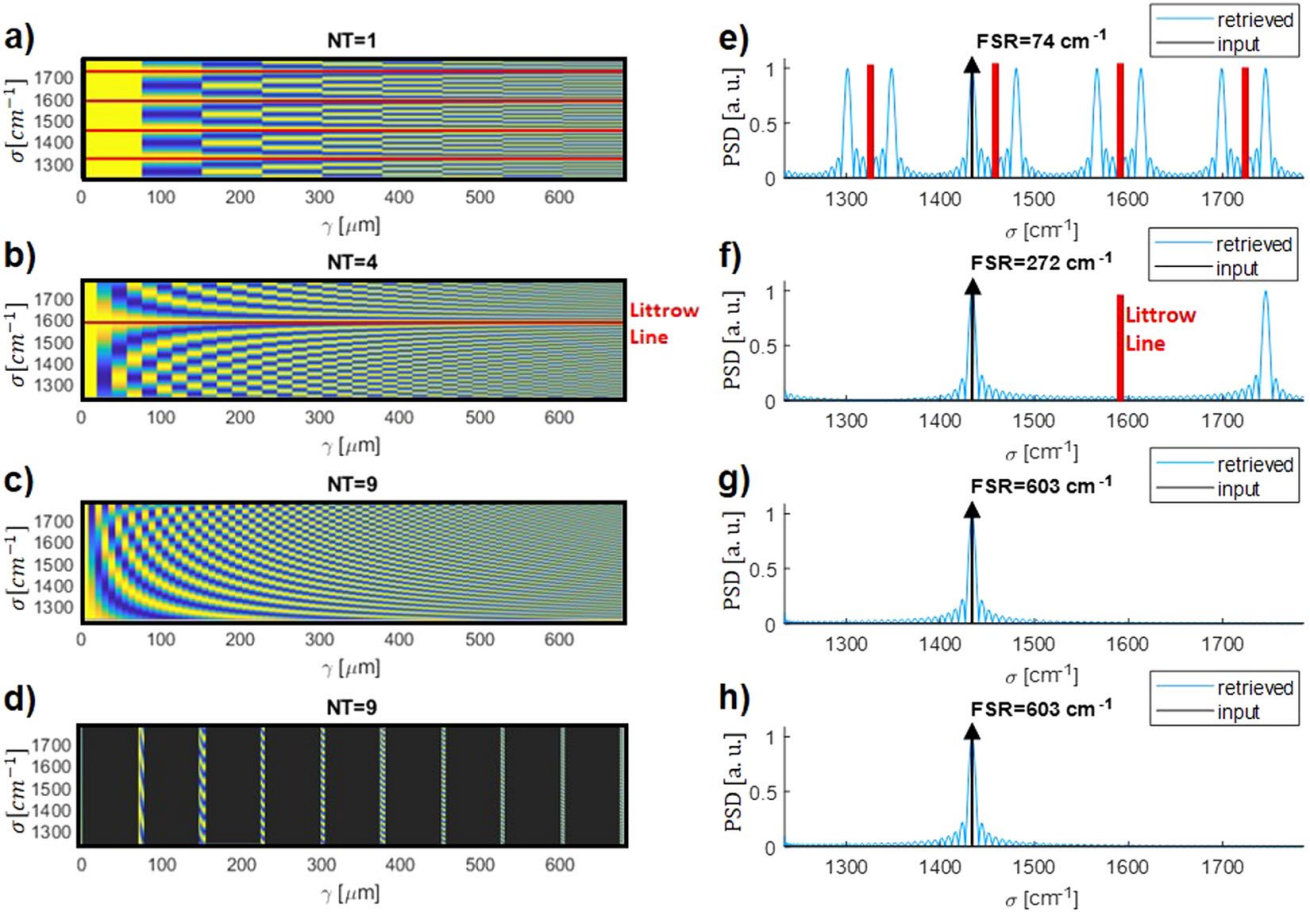
Retrieval process of a monochromatic input centered at 1434 cm^−1^ for different cases (**a**–**d**) Simulated interferograms ((**a**) no thermal tuning, (**b**,**c**) uniform sampling of the path delay with 4 and 9 different temperature steps, (**d**) non-uniform tuning of the path delay with 9 different temperature steps). (**e**–**h**) Power Spectrum Density (PSD) of the retrieved signal for the same examples. Littrow lines are reported in red.

To see the benefits of including a thermal tuning we plot in Fig. 2 the retrieval process of a monochromatic input centered at 1434 cm^−1^ (shown as a black arrow), in a 551 cm^−1^ bandwidth (5.6 *μ*m to 8.1 *μ*m) for different cases. Both the interferogram and retrieved spectrum are shown. Figure 2(a,e) illustrate the classical SHS-based FTS without thermal tuning. In this case the FSR is 74 cm^−1^, meaning that when trying to retrieve a signal in 551 cm^−1^, different repetitions of the input signal are obtained in the retrieved signal, in both sides of the different Littrow lines, that are separated by twice the FSR. In Fig. 2(b,c,f,g), the thermal tuning is implemented using a uniform sampling of the optical path delay. Δ*T*_*max*_ calculated using Eq. (5) with a maximum thermal increase of 54 K, which is an achievable value^33^. Δ*T*_*step*_ has thus been calculated using Eq. (4) for *N*_*T*_ = 4 and 9.

Interestingly, the increase of the FSR when *N*_*T*_ increases is clearly seen, and a proper retrieval is obtained for ${N}_{T}=9$, with a calculated of FSR of 603 cm^−1^.

Going further with this approach, instead of a discrete cosine transform this system can be seen as a generic linear transformation. Thus, it is possible to leverage the pseudo-inverse matrix method, or any other similar method that enables the user to reverse a linear transformation, to obtain any unknown input signal spectrum even in the case on nonlinear variations of *γ*. Therefore, it is possible to properly retrieve the input signal spectrum even if the optical delay is not sampled uniformly.

To demonstrate such property, in the last reported example (Fig. 2(d,h)), a non-linear *γ* sampling is tested. *N*_*T*_ is still equal to 9, as in the previous case, but Δ*T*_*max*_ and thus Δ*T*_*step*_ are reduced in comparison with the theoretical values. In this example $\Delta {T}_{step}=0.4\,K$ and $\Delta {T}_{max}=3\,K$.

As it can be seen from Fig. 2(h), even with such a non-uniform sampled setting it is possible to properly retrieve an input spectrum in a wavelength range from 5.6 to 8.1 *μ*m (551 cm^−1^), while the resolution is not affected.

**Figure 3:**
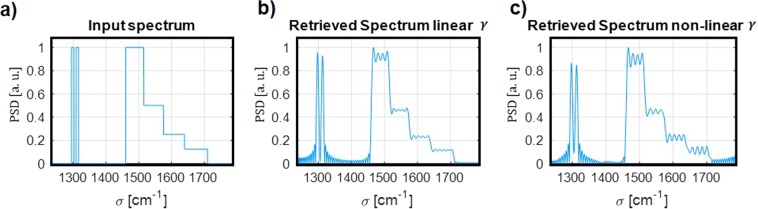
Retrieval of a polychromatic input spectrum. (**a**) Input Power Spectrum Density (PSD) in a wavelength range from 5.6 *μ*m to 8.1 *μ*m. (**b**) Retrieved spectrum with pseudo-inverse matrix method with linear *γ* sampling. (**c**) Retrieved spectrum with pseudo-inverse matrix method with non-linear *γ* sampling.

To illustrate this operation, the retrieval of the input spectrum shown in Fig. 3(a) is evaluated for ${N}_{T}=9$ and for both a linear and non-linear *γ* sampling. The theoretical normalized transformation matrix reported in Fig. 2(c,d) are used for the calculation. This input signal is formed by 2 narrow peaks separated by the theoretical resolution (13.4 cm^−1^), and with half-width at half-maximum corresponding to half of the theoretical resolution (6.7 cm^−1^). A broader signal with different levels is also considered on the right side of the spectrum, to check the behavior of the system with different power levels. The interferogram of the thermally-tuned FTS is calculated using such input signal spectrum and then the pseudoinverse matrix method is used to retrieve the input signal spectrum. This retrieved spectrum is reported in Fig. 3(b,c). This method clearly shows the capability to retrieve the broad signals amplitudes, as well as to distinguish the narrow peaks separated by the spectral resolution, within a wavelength range as broad as 551 cm^−1^ and potentially up to 603 cm^−1^. Interestingly there is almost no difference between the retrieved signals using the linear and non-linear sampling of *γ*.

In the previous examples the temperature increase has been set to only one arm of the MZI (the shortest in this example). Nevertheless, it is also possible to apply a temperature increment to both arms (longest and shortest arm) in a coordinate way, potentially getting the same linear increase of the optical path, but with a maximum temperature to be achieved on the chip divided by 2, i.e. 27 *K* in this case.

### Experimental results

**Figure 4:**
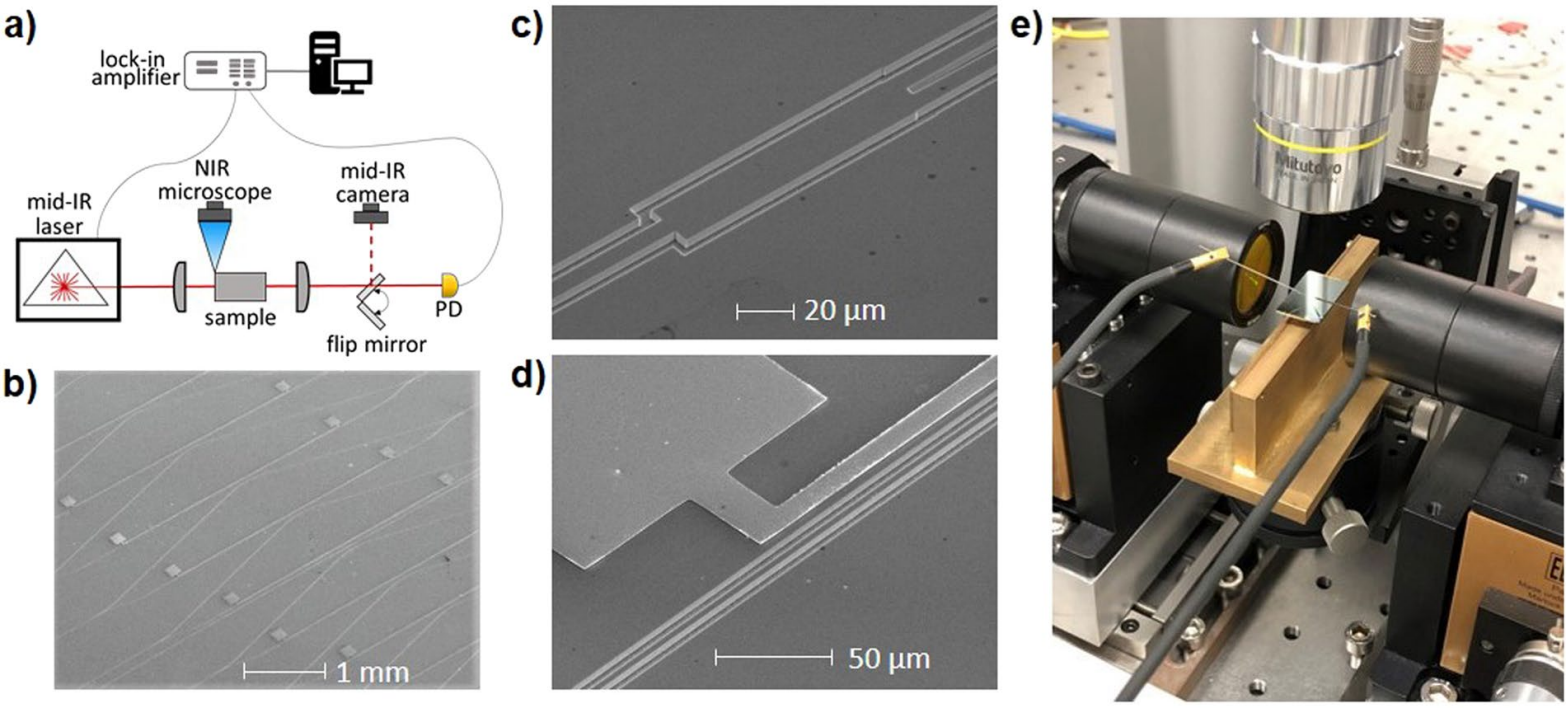
Characterization setup and Scanning Electron Microscope (SEM) images of the Fourier transform Spectrometer. (**a**) Scheme of the characterization setup. (**b**) General SEM picture of the MZI array, where the optical path is divided and recombined through asymmetric path lengths. Metallic contact paths placed at 12 *μ*m of the waveguides. MZIs are separated 50 *μ*m between each other. (**c**) SEM picture of a 1-by-2 MMI splitter. MMI dimension is 20 × 105 *μ*m. (**d**) SEM picture of the metallic path placed next to a straight waveguide. A wide path is used to facilitate the probe contact. (**e**) Picture of the device under test.

In Fig. 4(a) we can see the characterization setup schematic, where a tunable pulsed Quantum Cascade Laser (QCL) is coupled in and out of the the sample by aspherical lenses in free-space configuration. The mid-IR laser has a monochromatic linewidth below 1 cm^−1^ by datasheet specifications, which means that measured resolution will not be affected by the laser linewidth. Then, the output signal is collected by a Mercury Cadmium Telluride (MCT) broadband detector (PD), being the output signal amplified by a lock-in amplifier triggered with laser frequency (100 KHz). A mid-IR camera and a flipping mirror are used to ensure a correct coupling. In Fig. 4(b) the general overview of the tested sample structure is shown. The MZI array is based on a 4 *μ*m width and 4 *μ*m etching depth waveguides. A 1-by-2 Multi-Mode Interferometer (MMI) is used as splitter and combiner, as shown in Fig. 4(c). Gold contacts are placed next to the shortest MZI arm with two wide squared pads in order to facilitate the probe contact to induce thermal tuning (Fig. 4(d)).

The thermally-tuned FTS has been evaluated experimentally using a 10 MZI array with an Δ*L*_*step*_ of 18.8 *μ*m. The theoretical resolution and FSR of such 10 MZI array without any thermal-tuning are 14.6 cm^−1^ and 74 cm^−1^ respectively. Heaters have been added in the shortest arm of each MZI. A maximum temperature variation of 3 *K* has been applied on each MZI, which is much smaller than $\Delta {T}_{\max }=54\,K$ (deduced from Eq. (5)) to have a uniform sampling of the optical path delay. Moreover, due to technological issues the first 3 MZIs got damaged and are missing for this experiment. Thus, both the built-in phase variation in the MZI array and the temperature variation in each MZI create non-uniform sampling of the path delay.

Thermally-tuned FTS proof of concept thus relies on a 7 MZI-array, with non-uniform optical path delay sampling, where Δ*L* increases uniformly from 56.4 *μ*m to 169.2 *μ*m with 18.8 *μ*m increase. Interestingly, the flexibility and robustness of the thermally-tuned FTS operation even with such non-uniform path delay sampling will be shown in the following.

**Figure 5:**
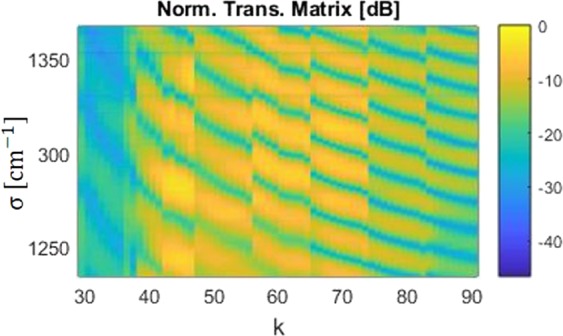
Normalized transmission matrix for 7 different MZIs with arm length difference from 56.4 to 169.2 *μ*m, at 9 different temperatures with a maximum increase of 3 K and measured from 7.3 to 8.1 *μ*m wavelength range.

As a first step, the transfer matrix is measured from 7.3 to 8.1 *μ*m wavelength (135 cm^−1^), with a 10 nm step and for ${N}_{T}=9$ points of temperature in each MZI, from Δ*T* = 0 to 3 *K*. Measurements have been done in TE polarization. The measured transmission matrix is plotted on Fig. 5.

**Figure 6:**
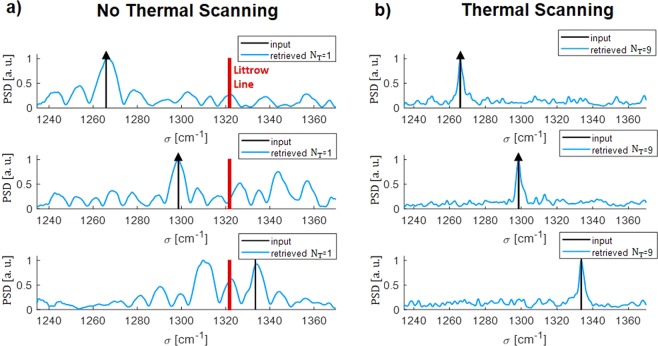
Experimental Power Spectrum Density (PSD) retrieval process in a wavelength range from 7.3 to 8.1 *μ*m. (**a**) Retrieved spectrum with non-thermal tuning for different monochromatic input positions. (**b**) Retrieved spectrum with ${N}_{T}=9$ and $\Delta {T}_{{\max }}\,K$, for different monochromatic input positions.

Based on the measured transfer matrix, signal reconstruction method has thus been applied for a monochromatic input centered at different wavelengths and represented as a black arrow in Fig. 6. A gaussian apodization window has been used to reduce truncation ripples.

The performance enhancement of the signal retrieval using the temperature scanning of the optical paths is clearly seen in Fig. 6. First, without temperature scanning the FSR is 74 cm^−1^, which is lower than the measured window spectral width. A repetition of the input signal is thus obtained symmetrically to the Littrow line. In comparison, the use of the thermal tuning allows an increase of the FSR up to 603 cm^−1^, which is much larger than the measured window and the signal spectrum can be correctly retrieved for the different cases. Furthermore, the thermal tuning approach yields a substantial increase in the signal-to-noise ratio, achieving a SNR of 7 dB. This improvement can be explained from the point of view of discrete Fourier transform and Parsival theorem. When a rectangular window is applied, as it is the case in our FTS, the expected frequency-domain squared power of a narrowband signal is proportional to the number of sampling points, while the square magnitude of the noise process remains constant^39^. Our approach increases the number of sampling points of the optical path delay by a factor of *N*_*T*_ (9 in the reporting case), thereby increasing the signal-to-noise ratio. Finally, the linewidth of the retrieved signal, and thus the resolution, without thermal tuning is degraded by the poor SNR. Then, the SNR enhancement in the thermal tuning case also lead to a linewidth reduction form 15 cm^−1^ to 5 cm^−1^.

## Discussion

It has been demonstrated in this work that it is possible to harness the thermo-optic effect to enhance the performance of on-chip FTS mainly improving the FSR. According to the Nyquist-Shannon theorem, the FSR is inversely proportional to two times the sampling interval (*γ*_*step*_). Here we propose to perform coarse sampling by MZI arm length unbalance and fine sampling by thermo-optical tuning of the path delay. This way we can achieve ultra-wideband retrieval capability, obviating the need of large number of MZIs. Interestingly, this effect can be used either to increase FTS performances or to reduce the number of MZI in the array to achieve given specifications, thus reducing the device footprint, or even both at the same time. With this approach it is thus possible to overcome or relax the scalability problem. Furthermore, since the method relies on a linear transformation which makes use of the pseudo-inverse matrix to retrieve the input spectrum, it has been shown that non-uniform sampling of the optical path delay can be used, thus reducing the required temperature variation and total power consumption.

Here, a mid-IR on-chip FTS with a resolution of 13.4 cm^−1^ was considered. The use of the thermal tuning allowed to increase the FSR up to 603 cm^−1^. This corresponds to more than 4-times enhancement of the FSR in comparison with the device reported by Q. Liu *et al*.^21^ with a reduction of the footprint by a factor of two, while keeping the theoretical resolution performance under 15 cm^−1^. We also foresee the possibility of using more performing retrieval algorithms, like elastic net method to retrieve the input spectrum.

In conclusion, these results provide to the best of our knowledge, the first experimental demonstration of on-chip MIR Ge-based FTS exploiting both spatial heterodyning and temperature tuning. The device resolution and FSR can be seamlessly designed by either thermally tuning or by the choice of the number of Mach–Zehnder interferometers. The maximum temperature increase or number of MZIs needed in the SHFTS can be further improved taking profit of pseudo-inverse retrieval or any other linear transformation method, and the resolution can also be enhanced by exploiting advanced numerical techniques, such as compressive-sensing, developed for FTS working in the near-infrared, at 1.55 *μ*m wavelength^25^. Finally, the on-chip integration of sensing circuits with the FT spectrometer could pave the way for future demonstration of robust, high-resolution, and cost-effective multi-target spectrometers covering an ultrawideband of the fingerprint wavelength range.

## Methods

### Fabrication and characterization

The device fabrication has been carried out by electronic lithography and Inductively Coupled Plasma etching. This process was used to demonstrate a first approach of FTS in mid-IR regime up to 8.5 *μ*m and was reported by Q. Liu *et al*.^21^. Then, a set of electrodes has been placed in the MZI, next to its shortest arm. Gold electrodes have been deposited by lift-off process.

The chip characterization has been carried out in a free space setup, using a tunable external cavity-based QCL. The optical beam is coupled into and out of the chip by a pair of aspherical lenses. Even though the device can carry both polarizations, it has been characterized on TE polarization. To set the thermal tuning, a current source equipment has been placed in the setup and connected to the chip by a pair of DC probes. In order to retrieve the simulated input spectrum, the Moore-Penrose inverse has been carried out in the pseudo-inverse matrix method.

### Thermal tuning

**Figure 7:**
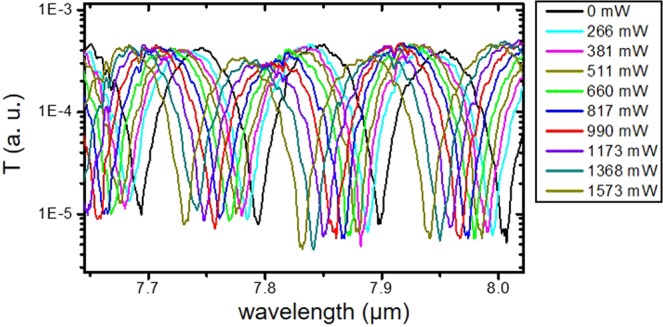
Transmission of MZI (arm length difference is 150.4 *μ*m) as a function of the wavelength for different dissipated power in the heater.

**Figure 8:**
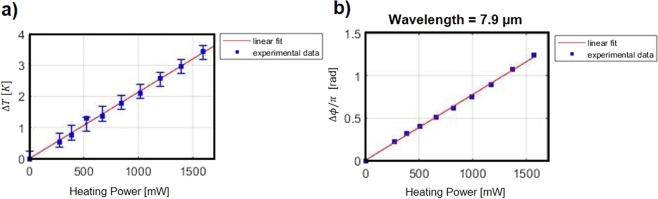
Thermal tuning performances. Experimental data in blue and linear fit in solid red. (**a**) Temperature variation in the waveguide as a function of the electrical power dissipated on the heater. (**b**) Phase shift variation as a function of the electrical power dissipated on the heater, at a wavelength of 7.9 *μ*m, deduced from Fig. 7 in red and linear fit in solid blue line.

The thermal tuning was first characterized by measuring the transmission of each MZI as a function of the dissipated power in the heater. The transmission of a MZI with Δ*L* = 150.4 *μ*m is reported in Fig. 7 as a function of the wavelength, for dissipated powers from 0 to 1.5 W. The dissipated power has been obtained by measuring the current applied on the device as function of the applied voltage. To achieve such thermal tuning, a 3.3 mm-long heater is placed next to the optical waveguide. The tracked phase variation centered at 7.9 *μ*m and the temperature variation inside the MZI waveguide deduced from the measurements are both reported in Fig. 8(a,b). The thermo-optic coefficient of the optical mode $\frac{\partial {n}_{g}}{\partial T}$ is supposed to be equal to the thermo-optic coefficient of Ge, assumed to be $4.1\times {10}^{-4}\,{K}^{-1}$.

An average heater efficiency of 2 K/W is deduced from the slope of the curve. This is the first demonstration of an active tuning on the graded Ge-rich SiGe waveguide platform. The efficiency is quite low, but the sample design was not optimized to enhance heater performance. For example, the distance between the optical mode center and the metallic ribbon was set to 12 *μ*m, as a conservative design to avoid any influence on optical losses in this first thermal-tuning demonstration. Further improvement of the heater design is envisioned to enhance the heater efficiency. Interestingly, despite a moderate heater efficiency, it is possible to achieve *π*-phase shift using a 3.3 mm heater length, as shown in Fig. 8(b).

### Mathematical analysis

For simplification, the mathematical analysis will be developed for a N MZI array with 1-by-2 MMI as splitter and combiner, but the analysis can be further generalized using any kind of multiport splitting/combining device. The optical path delay difference (*γ*) in an asymmetric thermally-tuned MZI can be written as in Eq. (6).

6 $$\gamma ={n}_{eff}\,\Delta L+{L}_{H}\,\Delta {n}_{eff,T},$$

where Δ*L* is the MZI arm length difference, *n*_*eff*_ is the mode effective index, $\Delta {n}_{eff,T}$ is the thermally induced effective index variation of the optical mode and *L*_*H*_ is the heater length (considering uniform temperature change along the heater length^40^). The effective index variation obtained by thermal tuning depends on the thermo-optic coefficient $(\frac{\partial {n}_{eff}}{\partial T})$ of the optical mode and on the temperature variation (Δ*T*) following:

7 $$\Delta {n}_{eff}=\frac{\partial {n}_{eff}}{\partial T}\,\Delta T,$$

The classical MZI array model can then be generalized to the thermally-tuned FTS by substituting ${n}_{eff}(\lambda )\Delta L$ by *γ*. The main relations are reported below.

Assuming lossless, perfectly balanced 50:50 splitters and combiner, the output power spectrum of a MZI ${p}_{out}(\sigma ,\gamma )$ is given by Eq. (8), where $\sigma ={\lambda }^{-1}$ is the wavenumber, and ${p}_{in}(\sigma )$ is the input spectral power.

8 $${p}_{out}(\sigma ,\gamma )=\frac{{p}_{in}(\sigma )}{2}[1+\,\cos (2\pi \sigma \gamma )],$$

Considering a broadband photodetector with flat wavelength response, we can write the measured MZI output power as in Eq. (9), where *P*_*in*_ is the integrated input power at the MZI^12^.

9 $${P}_{out}={\int }_{0}^{\infty }\,{p}_{out}(\sigma ,\gamma )\,d\sigma =\frac{1}{2}{P}_{in}+\frac{1}{2}\,{\int }_{0}^{\infty }\,{p}_{in}(\sigma )cos\mathrm{(2}\pi \sigma \gamma )d\sigma =\frac{1}{2}{P}_{in}+\frac{1}{2}I(\gamma ),$$

In the following, the measured output power of the MZI will be described by the interference term $I(\gamma )$, which can be easily obtained from measured ${P}_{out}(\gamma )$ as $I(\gamma )=2{P}_{out}(\gamma )-{P}_{in}$. The measured interference term is related to the input power density by:

10 $$I(\gamma )={\int }_{0}^{\infty }\,{p}_{in}(\sigma )cos\mathrm{(2}\pi \sigma \gamma )d\sigma ,$$

In practical implementation of thermally-tuned FTS, the spatial interferogram is discretized at $N={N}_{T}\times {N}_{L}$ equally spaced values of ${\gamma }_{k}\mathrm{(0}\le {\gamma }_{k}\le {\gamma }_{max})$, where *N*_*L*_ is the number of different MZI corresponding to different path lengths (from 0 to ${n}_{eff}\Delta L({N}_{L}-1)$ and *N*_*T*_ is the number of measurements at different temperatures for each MZI. Similar to reported by Florjanczyk *et al*.^12^, it can be shown that it is possible to retrieve the input spectrum ${p}_{in}(\widehat{\sigma })$ from the measured interferogram $I({\gamma }_{k})$ using a discrete Fourier cosine transform (Eq. (11)). To simplify the notation, the values of ${\gamma }_{i,j}$ are set in a row of ${N}_{T}{N}_{L}$ elements (*γ*_*k*_, k = 0, …, *N*_*T*_*N*_*L*_ − 1), concatenating each MZI at different temperatures. Moreover, because of the periodicity of the FTS transfer function which is described by its free spectral range (FSR), the wavenumber is shifted according to the Littrow line as $\widehat{\sigma }=\sigma -{\sigma }_{L}$^12^.

11 $${p}_{in}(\widehat{\sigma })=\frac{{\gamma }_{max}}{{N}_{T}{N}_{L}}{P}_{in}+2\frac{{\gamma }_{max}}{{N}_{T}{N}_{L}}\,\mathop{\sum }\limits_{k=1}^{{N}_{T}{N}_{L}}\,I({\gamma }_{k})cos\mathrm{(2}\pi \widehat{\sigma }{\gamma }_{k}),$$

The FTS performance is given by the signal retrieval resolution (*δσ*) and free spectral range (FSR). The FSR is related with the space between *γ*_*i*_ and the resolution by ${\gamma }_{max}$. Following a similar procedure reported by Florjanczyk *et al*.^12^, *δσ* and $FS{R}_{\sigma }$ (in wavenumber unit) of the thermally-tuned FTS are given in Eqs (1) and (2), where $\Delta {n}_{eff,T,max}$ is the maximal effective index variation induced by the heater and $\Delta {L}_{max}=\Delta L({N}_{L}-\mathrm{1)}$. Interestingly they can be compared with the ones of classical FTS given by Eqs (12) and (13).

12 $$\delta \sigma =\frac{1}{\Delta {L}_{max}\,{n}_{g}},$$

13 $$FS{R}_{\sigma }=\frac{1}{\Delta {L}_{max}\,{n}_{g}}\frac{{N}_{L}}{2},$$

Since the thermo-optic coefficient can be considered constant with the wavelength^24^, it can be noted that in previous equations the effective index variation respect to the wavelength remains constant with the temperature.
